# Decrypting Financial Markets through E-Joint Attention Efforts: On-Line Adaptive Networks of Investors in Periods of Market Uncertainty

**DOI:** 10.1371/journal.pone.0133712

**Published:** 2015-08-05

**Authors:** Niccolò Casnici, Pierpaolo Dondio, Roberto Casarin, Flaminio Squazzoni

**Affiliations:** 1 Department of Clinical and Experimental Sciences, University of Brescia, Brescia, Italy; 2 School of Computing, Dublin Institute of Technology, Dublin, Ireland; 3 Department of Economics, University Ca’ Foscari, Venice, Italy; 4 Department of Economics and Management, University of Brescia, Brescia, Italy; University Toulouse 1 Capitole, FRANCE

## Abstract

This paper looks at 800,000 messages on the Unicredit stock, exchanged by 7,500 investors in the Finanzaonline.com forum, between 2005 and 2012 and measured collective interpretations of stock market trends. We examined the correlation patterns between market uncertainty, bad news and investors' network structure by measuring the investors' communication patterns. Our results showed that the investors' network reacted to market trends in different ways: While less turbulent market phases implied less communication, higher market volatility generated more complex communication patterns. While the information content of messages was less technical in situations of uncertainty, bad news caused more informative messages only when market volatility was lower. This meant that bad news had a different impact on network behaviour, depending on market uncertainty. By measuring the investors' expertise, we found that their behaviour could help predict changes in daily stock returns. We also found that expert investors were more influential in communication processes during high volatility market phases, whereas they had less influence on the real-time forum's reaction after bad news. Our findings confirm the crucial role of e-communication platforms. However, they also show the need to reconsider the fragility of these collective intelligence systems when under external shocks.

## Introduction

In the Internet era, investors share information through online communication platforms (e.g., [1], [2] and [3]). Professional traders and non-professional investors consider them a means to cope with risky investment decisions in complex market environments where the signalling function of prices alone cannot help investors understand what is going on (e.g., [4] and [5]). Recently, empirical finance studies have emphasized the importance of understanding how these information sharing platforms can generate relevant knowledge, which is eventually incorporated into market dynamics (e.g., prices and stock returns).

For instance, [6] examined the relation between on-line messages and stock returns and trading volume on the *RangingBull*.*com* forum. These authors found that abnormally high message activity correlated with abnormal stock returns. This result was also found by [7], who examined the effect of more than 1.5 million messages posted on Yahoo! Finance and Raging Bull. They found that stock messages helped to predict market volatility on a daily basis, even within the same trading day. Their results showed that higher message postings predicted subsequent negative returns and that disagreement between the posted messages was associated with increasing trading volume. Significant correlations were also found when looking at more general social media, such as Twitter. Indeed, [8] found that Twitter mood predicted more than 80% of daily volatility of the closing value of the Dow Jones Industrial Average. This was also found for Google query volumes for search terms related to finance, which could anticipate complex stock market trends [9].

These studies investigated the potential of knowledge generating mass collaboration processes in influencing financial markets and suggest a complex adaptive systems perspective to financial markets (e.g., [10] and [11]). However, they only looked at aggregate dimensions, without entering into the “black box” of the network structure of communication between investors. Furthermore, they did not consider the heterogeneous roles that individuals can play in communication processes. These are essential to understand how investors cope with uncertainty by developing knowledge collaboratively and how they find reliable information sources from anonymous interactions (e.g., [12] and [13]).

The aim of our paper was to fill this gap by examining correlation patterns between stock returns, information exchange and investors’ network configuration, also measuring the network structure of the joint-attention effort by investors. We focused on the *finanzaonline*.*com* forum, the leading Italian financial online community, which includes more than 150,000 active investors. Established at the end of 1999 by *Brown Editore*, an independent and highly influential publishing company specializing in high quality economic and financial information, *finanzaonline*.*com* immediately became the main information and communication online platform for Italian investors. The website provides real time data and financial analysts’ reports and hosts the most popular peer-to-peer communication platform for investors in Italy. For instance, last year the forum had more than 750 active daily users and more than 3,400 messages posted per day. The average monthly number of forum visitors is more than one million. Communication is organized through threads, which are structured in five rooms as follows: live meetings, operative finance discussions, financial in-depth analysis, free discussions and customer support.

From the time series of messages, we extracted 800,000 posts on the Unicredit stock between 2005 and 2012. We focused on this stock as it was extremely volatile and attracted considerable attention in the press and the media. Unicredit is one of the most recognized banks both in Europe and worldwide, with its headquarters in Milan. It has more than forty million customers and operates in twenty-two different countries. Its main markets are Italy, Austria, Southern Germany, Switzerland, Central, and Eastern Europe. The company is listed on the Milan Stock Exchange (ISIN code: IT0000064854, alphanumeric code: UCG) and is included on the FTSE MIB stock market index.

Our aim was to measure the forum’s network structure and dynamics in order to understand how investors cope with uncertainty and bad news by creating self-organized, distributed, decentralized joint-attention processes. Well-studied in evolutionary psychology as a feature of children’s social learning (e.g., [14], [15] and [16]), the importance of joint-attention has been largely underestimated when looking at complex human behaviour. We hypothesised that online forums and e-communication platforms could be viewed today as a social experiment of “e-joint attention”, i.e., a social process through which a multitude of individuals focus on relevant matters collectively, anonymously and at a distance, by combining different interpretations and learning from ‘observing’ interpretations favoured by e-communication platforms. This is essential to understand behaviour of investors in periods of ubiquitous, global, real-time finance and (ontological, semantic, operational) market uncertainty (e.g., [17]).

First, to measure this phenomenon empirically, we distinguished periods of high and low market volatility and analysed the network behaviour in these situations. We then isolated good and bad market news to consider how the network was capable of reacting in real-time. We assumed that low and high market volatility was a proxy of good or bad news on markets and that market prices reflected all information available to investors, as common in finance. Distinguishing between good and bad news, i.e., low or high market volatility, was essential to look at the potential asymmetric reaction of investors (e.g., [18], [19] and [20]). We then mapped the daily network structure of communications dealing with Unicredit stock among investors by identifying five daily variables: (i) network activity, in terms of the number of nodes, edges and messages, (ii) network stability, in terms of daily investors' turnover and refresh of ties, (iii) network fragmentation, in terms of network modularity [21], (iv) the information content of messages exchanged in the network and (v) the presence and role of the network of expert investors (hubs).

In order to examine the dynamic relation between the network topology and Unicredit stock returns, we used a Bayesian Vector Autoregressive Model (VAR) that looked at the correlation between the Unicredit stock returns and forum network dynamics, by using the same groups of network variables that we built for the threshold analysis. It is important to note that Bayesian VAR models have been widely used in multivariate time series analysis to examine the dynamic relations between different economic variables (e.g., [22], [23] and [24]). Bayesian VAR are flexible models that permit to deal with potential over-parameterization problems, which are typical when vectors of large or moderate dimensions are considered (e.g., [25], [26] and [27]). Given that we aimed to investigate the relation between network topology and markets and especially its structural stability under different market conditions (regimes), we considered the Bayesian Markov-switching VAR models (MS-VAR) which allowed us to consider VAR parameters that change over time. The time-varying autoregressive coefficients helped us to consider possible structural instability in the stock return and forum network dynamics. Time-varying volatilities and correlations between all variables permitted us to check for heteroschedasticity in measurement errors. The resulting nonlinear model also allowed us to deal with the excess of kurtosis and skewness observed in the time series. In order to identify volatility regimes, we followed a standard approach by using parameter identification constrains (e.g., [28]). We assumed that the variance parameter for the Unicredit stock return’s equation increased with the regime index. This allowed us to identify the first regime as the low volatility period and the last one as the highest volatility period. In this way, the inference procedure permitted to separate the observations in different periods with different volatility levels.

Our results showed that uncertainty and market bad news increased the joint-attention effort of investors and that the network structure varied depending on market situations. On the one hand, both high volatility and bad news triggered higher participation and more complex communication patterns. On the other hand, high volatility caused less informative messages. Furthermore, by measuring the degree of expertise of all investors, we found that expert investors communicated more in periods of high volatility and after market bad news. The distribution of experts throughout the system could ideally be seen as a source of resilience and information redundancy, which are typically associated with complex system behaviour in turbulent and uncertain periods. However, while during high volatility, the network rallied around expert opinion, these experts were less influential for the system’s real-time reaction after market bad news. This was because market volatility implies iterated patterns of communication through which investors can efficiently self-organise around experts’ opinions, whereas external shocks, i.e., bad news, requires real-time reactions that these decentralized, self-organised systems can perform only inefficiently. Finally, we found that independent of volatility, the forum network included knowledge which could predict stock market daily returns, thus confirming the importance of these online communication platforms for information sharing (e.g. [8] and [9]).

The structure of the paper is as follows: The second section describes the available data, while the third illustrates the methods used to extract the volatility regimes, map the forum network, estimate the link between market and forum dynamics and measure investors’ expertise. The fourth presents the results. The fifth discusses the main findings by suggesting an explanation of network behaviour in terms of e-joint attention processes.

## Data

We focused on the Italian market sub-section of the operative finance room. One of the main topics discussed on the forum was the Unicredit stock, which attracted more than 7,000 investors. We extracted all posts in all Unicredit stock threads between 2005 and 2012, a total of more than 800,000 messages. We then considered the communication patterns among investors posting on the forum as a social network and mapped the network structure on a daily basis by creating a longitudinal series of communication graphs. We considered all investors posting at least one message on the Unicredit thread as nodes and every message directed node-by-node through the "quoting" mechanism as edges. This helped us to obtain a time series of directed and weighted networks. We then analysed the temporal changes of the network structure through five variables: (i) network activity, (ii) network stability, (iii) network fragmentation, (iv) information content and (v) the role of network hubs.

**Fig 1:**
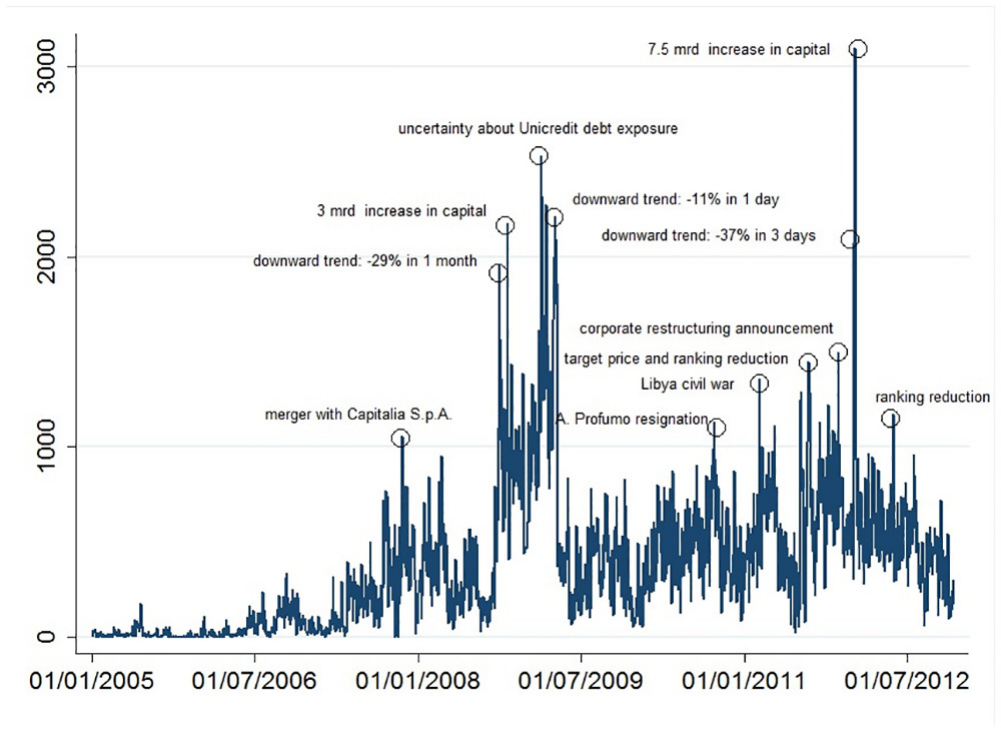
The daily number of messages on Unicredit from 2005 to 2012 (*M*_*t*_).

First, we considered the volume of messages (*M*_*t*_), the number of nodes (*N*_*t*_) and the number of edges (G_*t*_) as daily indicators to measure the information exchange activity on the Unicredit stock in the forum. Fig 1 shows the time series of messages about Unicredit from 2005 to 2012. It is worth noting that messages significantly increased in the period 2005–2007, when the bank expanded rapidly both at an international (i.e., merging with HVB and BA-CA groups) and national level (i.e., merging with Capitalia). The series showed high volatility with three peaks in late 2008 and 2011. At the end of September 2008, the Unicredit stock fell 29% and the board of directors decided to increase capital. The negative trend for the stock continued until March 2009. Given the high volatility of the stock at the end of 2011, the board of directors undertook another capital increase at the beginning of 2012. In this period, Unicredit stock lost more than 35% of its value in only three days (one of the last peaks in Fig 1 was associated with this event).

The stability of the network over time was measured by the daily turnover rate of the nodes and the refresh rate of the edges (see Methods for detail). By following the modularity algorithm of community detection suggested in [21], we measured the strength of the division of the network into communities (D_t_) and the number of communities (B_t_), which represented network fragmentation into sub-structures. These sub-structures could be considered circumscribed groups of discussion about a specific topic related to Unicredit, e.g., the stock market trend, market rumours on Unicredit or future perspectives of the banking sector. While networks with high modularity coefficients and high numbers of communities indicated that investors communicated about different topics in different groups, networks with low modularity coefficients and a small number of communities indicated that investors communicated about only a few similar topics more horizontally.

Furthermore, we looked at the content of messages by distinguishing those including valuable information on markets from those reporting information not related to markets. While the former included technical analysis (X_t_), fundamental analysis (stock fundamentals) (U_t_), news (market news) (W_t_), strategies (disclosure of trading opinions, sentiment and strategies) (S_t_), the latter included messages that did not fall in one of the previous categories or were out-of-topic (C_t_) (see S1 File for detail). We calculated a daily value of financial content of information (I_t_) by dividing the daily number of messages of the first type over the total number of messages posted in the forum. The higher the value of I_t_, the more the average amount of financial content was stored daily on the forum.

Given the importance of considering the potential presence and role of opinion leaders on the forum network, we built a model that ranked each investor according to their investment experience and trustworthiness. The model included five features: (i) communication activity of the investor on the forum, (ii) the longevity of their active presence on the forum, (iii) the regularity of their communication activity over time, (iv) the pertinence of the information content of their messages, and (v) the investor’s influence on the forum (see Methods for detail). We then measured the daily number of expert investors posting on Unicredit (*T100*_*t*_) and their relative importance for communication dynamics of the forum (*DIN*_*t*_). In order to estimate the importance of any investor, we measured their in-degree value as a proxy of their structural prestige in the forum [29]. The variable *DIN*_*t*_ indicated the difference between the average in-degree of the most expert and that of less experienced investors.

Finally, we calculated the Unicredit stock daily closing prices, (*p*_*t*_), of the Unicredit stock by gathering data provided by the website *Borse*.*it*. We defined the daily stock returns (*r*_*t*_) as the logarithm of the stock price at time *t*, minus the natural logarithm of the stock price at time *t-1* as follows:

$${\text{r}}_{\text{t}}=\text{ln}\left({\text{p}}_{\text{t}}\right)-\text{ln}\left({\text{p}}_{\text{t}-1}\right)$$ (1)

In order to examine the complex relation between market and forum, we conducted two complementary analyses. First, we focused on network behaviour under different volatility regimes of the Unicredit stock (Tables A, B and C in S1 File). By considering the volatility as a proxy of market uncertainty, we focused on the effect of the different uncertainty phases on communication patterns. Secondly, we built a Vector Autoregressive Model (VAR) to look at bad news and investigate the impact of external shocks on communication patterns (see Methods for details). We considered market decreases as proxy of bad news and market increases as proxy of good news. The data showed that the very low volatility market phase corresponded with the first period when Unicredit started to be discussed on the forum. In order to avoid being excessively influenced by this initial period, we especially focussed on regimes of high and low volatility when the stock had more interesting dynamics and a more mature process of communication was reached by forum investors.

## Methods

Our dataset included all messages on Unicredit exchanged by 800,000 investors between 2005 and 2012, with the threat title and ID, the ID of the forum user, the title and content of the message, the day and hour of the message. The ethics committee of the University of Brescia approved this retrospective study. Participant records/information was anonymized and de-identified prior to analysis. The stability of the network was measured by the daily turnover rate of the nodes and the refresh rate of the edges. The first indicator was the inlet/outlet flow of active investors compared with the previous day (*F*_*t*_) and was calculated as follows:

$$F_{t}=\frac{\left(\;U^{+}{}_{t}-\;U^{-}{}_{t}\right)}{\left[0.5\left(N_{t}+\;N_{t-1}\right)\right]}$$ (2)

where *U* ^+^_*t*_ was the total number of new active investors compared with the previous day (in-let flow) and *U* ^-^_*t*_ was the number of investors who left the forum compared with the previous day (out-let flow). The investors’ turnover reflected the stability of the node structure over time: the higher *F*_*t*_ was, the more the network changed day-to-day. Similar to (*F*_*t*_), the refresh rate of ties (P_t_) was calculated as follows:

$$P_{t}=\frac{\left(\;T^{+}{}_{t}-\;T^{-}{}_{t}\right)}{\left[0.5\left(N_{t}+\;N_{t-1}\right)\right]}$$ (3)

where *T* ^+^_*t*_ was the total number of new ties compared with the previous day and *T* ^-^_*t*_ was the number of broken ties compared with the previous day. As before, P_t_ indicated the stability of the tie structure over time; the higher P_*t*_ was, the more the network changed from day-to-day.

As for the presence and role of more competent investors in the network, we built a model that ranked all forum investors according to their expertise. The model included five features as follows: (i) the communication activity of investors, (ii) the longevity of their active presence in the forum, (iii) the regularity of their communication activity over time, (iv) the pertinence of information content of their messages, and (v) investors’ influence on the forum (Tables E, F and G and Fig A in S1 File).

As regards the choice of network variables to specify in our MS-VAR, in order to avoid multi-collinearity problems, we only included the number of users (*N*_*t*_) from the first group of variables, given the high correlation of *N*_*t*_ with other group variables, i.e., *M*_*t*_ and *G*_*t*_. Similarly, considering the last set of variables, we only included the synthetic indicator of financial content of messages *I*_*t*_, as this correlated closely with all other variables. In order to control for the financial market effect of the daily log-return on the FTSE MIB All share market index at time t-1, only *b*_*t*_, was included as a covariate. The following vectors of variables

$$x_{\text{t}}=\left(1,b_{t}\right)\prime$$ (4)

and

$$z_{\text{t}}=\left(N_{t},F_{t},P_{t},D_{t},B_{t},T100_{t},DIN_{t},I_{t}\right)\prime$$ (5)

were included in a *n* = 9 dimensional Bayesian MS-VAR model, which was defined as follows:

$$\left(\begin{array}{c} z_{\text{t}}\\ {\text{r}}_{\text{t}} \end{array}\right)=\text{A}\left({\text{s}}_{\text{t}}\right)x_{\text{t}}+\text{B}\left({\text{s}}_{\text{t}}\right)\left(\begin{array}{c} \begin{array}{c} z_{\text{t}-1} \end{array}\\ {\text{r}}_{\text{t}-1} \end{array}\right)+\varepsilon_{\text{t}}$$ (6)

**ε**_**t**_~*N*_n_(**0**,Σ(s_t_)), independently distributed for *t* = 1,…,*T*, with:

$$A(s_{t})\;=\;\sum_{k\;=\;1}^{K}A_{k}I(s_{t}\;=\;k),\;B(s_{t})\;=\;\sum_{k\;=\;1}^{K}B_{k}I(s_{t}\;=\;k),\;\Sigma (s_{t})\;=\;\sum_{k\;=\;1}^{K}\Sigma_{k}\;I(s_{t}\;=\;k)$$

where A_k_, k = 1,…,K, was a sequence of n×m matrices of regime-specific coefficients, B_k_, k = 1,…,K, was a sequence of n×n matrices of regime-specific autoregressive coefficients and Σ_k_, k = 1,…,K, was a sequence of covariance matrices. The indicator function *I*(*s*_*t*_ = *k*) took value 1 if *s*_*t*_ = *k* and 0 otherwise. We assumed that the model parameter was driven by a latent random process *s*_*t*_, t = 1,…,*T*, which took a value 1, 2 or 3. We assumed that this process was a homogenous Markov chain with transition probabilities *P*(*s*_*t*_ = *j|s*_*t-1*_ = *i*) = *p*_*ij*_, i.e., the probability to be in the regime *j* at time *t* depended solely on the previous value of the chain, i.e. *s*_*t-1*_ = *i*, constant over time. In order to identify the regimes as increasing volatility regimes we assumed the identification constrain *σ*_11,1<_ *σ*_11,2<_ *σ*_11,3_, where *σ*_11,k_ indicated the volatility of the Unicredit return equation in the regime *k*, i.e., the first element of the main diagonal of Σ_k_. This identification constrain was not based on a statistical analysis of the output of the inference process, but reflected our hypothesis of the relation between financial market and forum (e.g., [28]). Nevertheless, this constraint was effective to separate observations in three groups with different volatility and VAR coefficient values. Moreover, given that it turned out that the first regime corresponded to the initial part of the sample across different specifications of the number of regimes (see S1 File), we labelled the first regime as “initial regime”. The regimes 2 and 3 were labelled respectively as “low” and “high” volatility regimes as they corresponded to an increasing level of volatility. Finally, it is worth noting that the specification of the Bayesian model was completed before the distribution of model parameters. Following [30], we assumed a conjugate normal-Wishart prior distribution

$${((vec\;A}_{k})',\;(vec\;B_{k})')'∼N_{n^{2}+2n}\;(0,\Upsilon ),\;\Sigma_{k}∼W_{n}\;(\nu ,S),\;(p_{i1},\ldots ,p_{iK})∼Dir(\alpha_{i1},\ldots ,\;\alpha_{iK})$$

where *vec* indicated the vectorization operator, using the hyper-parameter setting that was suggested in [24] and [30]. More specifically, as regards to the main diagonal of the matrix γ, we assumed that the conditional standard deviation of the coefficient on lag *l* of the variable *j* in the equation *i* was $\lambda_{0}\lambda_{1}/(\sigma_{j}l^{\lambda_{3}}$), while the conditional standard deviations of the intercept and the exogenous variables were *λ*_0_ *λ*_4_ and *λ*_0_ *λ*_5_, respectively. Note that the hyper-parameters *σ*_*j*_ were choosen as the sample standard deviations of residuals from univariate autoregressive models fit to the individual series in the sample. The hyper-parameters *λ*_0_ and *λ*_1_ were set to 1 and controlled the tightness of the prior (discounting of prior scale) and the deviation or tightness of the prior around the AR(1) respectively. The hyper-parameters *λ*_3_, *λ*_4_ and *λ*_5_ were set to 1 and controlled the lag decay, the standard deviation around the intercept and standard deviation around the exogenous variable coefficients, respectively. As regard to the prior on the sum of the autoregressive coefficients, we used a dummy observation approach and looked at a correlation between all coefficients on a given variable in a given equation by adding a set of dummy observations to the data matrix. The values were given by the average of the initial values of the dependent variable multiplied by *μ*_5_. When *μ*_5_→∞, the model generated a form similar to differenced data. In our application, the series had no unit roots. So, we set the hyperparameter *μ*_5_ = 0.1. The dummy initial observations component introduced correlations in prior beliefs about all coefficients of a given equation. When *μ*_6_→∞, the model generated a form in which all variables were stationary, i.e., equal to the sample averages of the initial conditions. Therefore, we set *μ*_6_ to 0.1. For the *i*-th row of the transition matrix, we assumed a Dirichlet prior distribution with the parameter vector (*α*_1_,…,*α*_*K*_), which was given by the i-th row of the matrix 100*I*_*K*_+2*ιι′* where *ι* was the K-dimensional unit vector. The prior degrees of the freedom parameter *v* of the Wishart matrix was set to 25.

**Fig 2:**
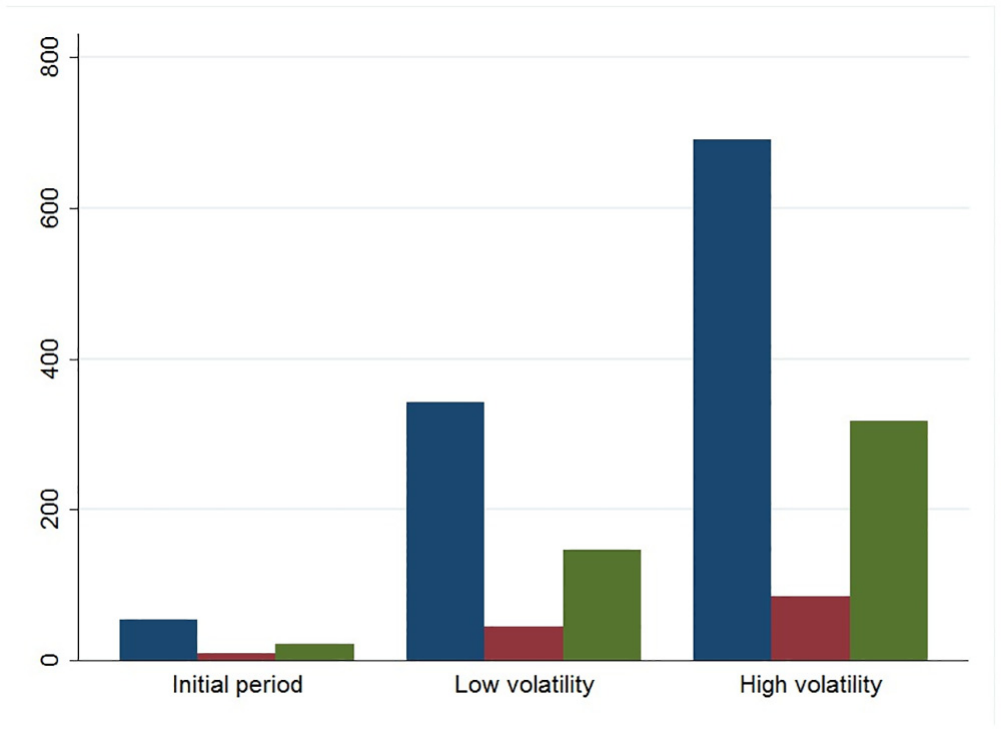
Unicredit stock log-return, *r*_*t*_, series (blue line, left axis) and the filtered regime of volatility *s*_*t|t*_ (red line, right axis).

Tab. 2 shows the estimates (posterior means) of the autoregressive coefficients in the three regimes. Finally, a key quantity in the estimation, which was also a useful output of the inference procedure, was the probability to be in a low-volatility regime condition on the information available up to time *t*. This was called a filtered probability and is denoted with ξ_*kt+t*_ = *P*(s_t_ = *k*|*r*_1_,…,*r*_*t*_,**z**_1_,…**z**_*t*_) (e.g., [31] and [28]). The filtered probabilities ξ_*kt+t*_, *k* = 1,2,3, were obtained by the Hamilton-filter recursions. The filtered hidden state was obtained as s_*t|t*_ = arg max{ξ_*kt+t*,_ *k* = 1,2,3}. Fig 2 shows the stock return series (blue line, left axis) and the filtered regime of volatility s_*t|t*_ (red line, right axis).

## Results

**Fig 3:**
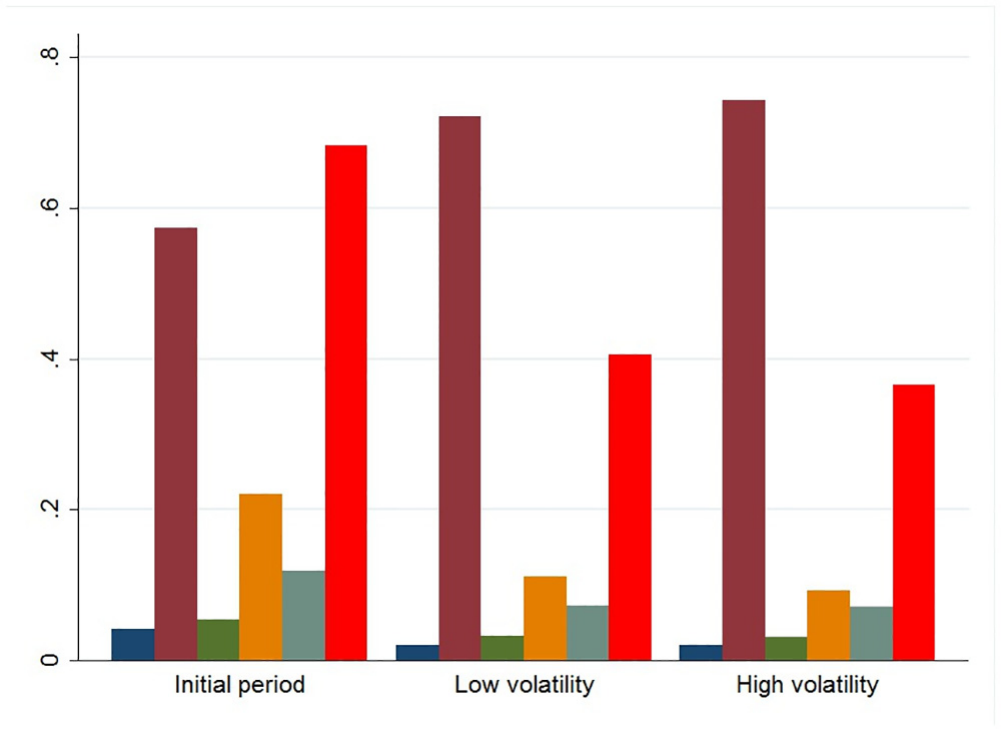
The degree of investors’ participation in the three volatility phases. Blue bars indicate the average number of messages, purple bars show the average number of investors active in the forum, green bars show the average number of ties between the investors.

**Fig 4:**
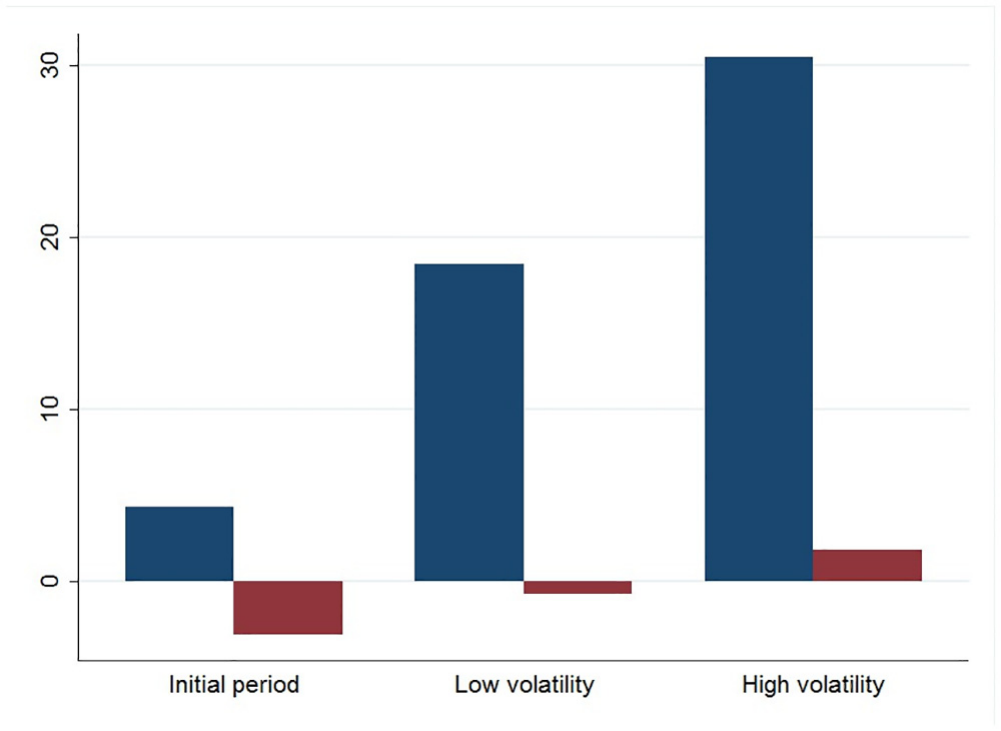
The presence and influence of expert investors in the volatility regimes. Blue bars indicate the number of active expert investors, while purple bars show the difference between the average in-degree of more and less expert investors.

**Fig 5:**
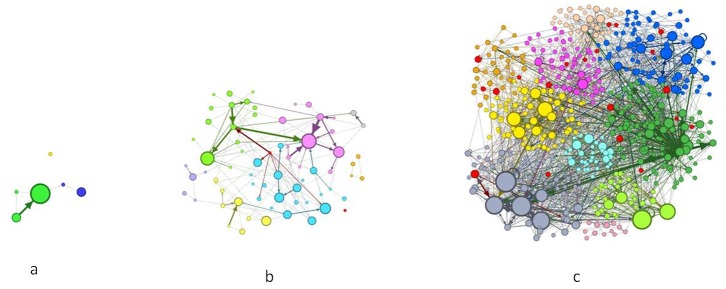
The information content of the messages in the three volatility phases. Blue bars show the average of fundamental analysis messages, purple bars the average of the residual content messages, green bars the average of the news reporting messages, orange bars the average of technical analysis messages, grey bars the average of strategy messages and red bars indicate the average value of the synthetic indicator of financial content.

Our initial results indicate that the network activity was strongly influenced by market volatility regimes. In situations of high volatility, more investors were active on the forum and communicated more by establishing more ties with each other (see Fig 3). Higher volatility also attracted higher participation by expert investors (see Fig 4), whose social prestige tended to increase during high volatility situations. Furthermore, high volatility decreased the information content of messages, which tended to systematically change from more technical and analytical to more residual content when volatility increased (see Fig 5). On the other hand, market volatility did not change the network’s structure, i.e., stability and fragmentation. It is worth noting that the number of sub-groups was higher in situations of high volatility, also due to the higher number of investors involved, but the strength of the inter-group separation did not change.

The initial period was different compared to other volatility regimes. In this case, the network attracted fewer investors, there was less information sharing, communication patterns were flatter, with fewer sub-groups and less separation, but messages between investors had more information content (Tables H, I and L and Fig B in S1 File).

**Fig 6:**
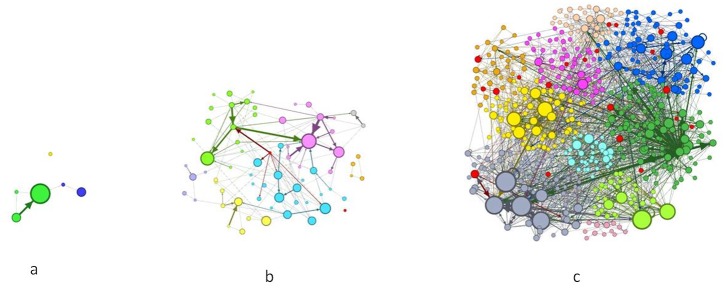
The communication network during the three volatility regimes. Initial period: day 31/01/2006 (a). Low volatility: day 02/01/2012 (b). High volatility: day 09/01/2012 (c).

Fig 6 shows the forum network during the three volatility regimes, i.e., the initial period, low and high market volatility respectively. We selected these three days as important market news was released during them. These snapshots were taken given that the stronger the link between two investors, the thicker the graph’s ties would be. The node size was proportional to the investor’s in-degree. The difference of node colour indicates a different modularity class. The red nodes represented the most competent investors as calculated by our model. Results confirmed that the forum was more active during periods of higher volatility and that more competent investors were more present on the forum during high volatility phases.

We wanted to understand how the network reacted to market news in real time and whether the forum included useful information for predicting market behaviour. We therefore calculated the impact of bad and good market news of the previous day on the network topology in each volatility regime using a Markov switching VAR (MS-VAR) framework (see Methods for details). Table 1 shows the relation between forum variables and Unicredit returns under the three different volatility conditions. The estimated coefficients of each equation of the MS-VAR model are shown in different columns.

**Table 1:** 

a) Initial period (*s*_*t*_ = 1)										
	Intercept	*r*_*t*-1_	*N*_*t*-1_	*F*_*t*-1_	*P*_*t*-1_	*D*_*t*-1_	*B*_*t*-1_	*T*100_*t*-1_	*DIN*_*t*-1_	*I*_*t*-1_
*r*_*t*_	0.00	**0.00**	0.01*	0.00	0.00	0.00	0.01*	0.00	0.01*	0.00
*N*_*t*_	3.30*	**-45.19** *	0.68*	0.14	-0.22*	-6.13*	0.12	1.14*	-0.06*	0.76*
*F*_*t*_	0.72*	**-7.01** *	0.01*	-0.08*	-0.12*	-0.68*	-0.01	-0.14*	0.02*	0.12
*P*_*t*_	0.27*	**-8.20** *	0.00	0.01	-0.13*	-0.74*	-0.04*	0.07*	0.01*	0.38*
*D*_*t*_	0.19*	**0.25** *	0.01*	0.00	0.01*	0.06*	0.00	-0.03*	0.01*	0.02*
*B*_*t*_	1.37*	**-5.98** *	0.06*	0.01	-0.04	-0.37	0.16*	0.08*	-0.03*	0.46*
*T*100_*t*_	0.03*	**-0.56** *	0.00*	0.01	0.00	0.10*	-0.01*	0.04*	0.00*	-0.01
*DIN*_*t*_	0.04	**9.04** *	0.01*	-0.06	0.05	0.43	-0.24*	-0.31*	0.64*	-1.02*
*I*_*t*_	0.58*	**-0.56** *	0.00	-0.01	0.03*	0.06	-0.03*	-0.02*	0.00	0.25*
b) Low-volatility period (*s*_*t*_ = 2)										
	Intercept	*r*_*t*-1_	*N*_*t*-1_	*F*_*t*-1_	*P*_*t*-1_	*D*_*t*-1_	*B*_*t*-1_	*T*100_*t*-1_	*DIN*_*t*-1_	*I*_*t*-1_
*r*_*t*_	-0.01*	**-0.04** *	0.01*	0.00	0.00	0.01*	0.01*	0.00	0.01*	0.00
*N*_*t*_	21.42*	**-7.35**	0.57*	-4.63*	3.32*	3.12	0.57*	-0.32*	0.02	-13.00*
*F*_*t*_	0.63*	**-1.02** *	0.00*	0.10*	-0.18*	-0.37*	-0.02*	0.01	0.00	-0.13*
*P*_*t*_	0.68*	**-1.47** *	0.00*	0.33*	-0.37*	-0.01	-0.01	-0.01	0.01	-0.10
*D*_*t*_	0.14*	**-0.06**	0.00	0.01	0.00	0.42*	0.00	0.01*	0.00	0.06*
*B*_*t*_	3.61*	**-0.41**	0.02*	-0.26*	0.24*	2.45*	0.13*	0.01	-0.01*	-0.93*
*T*100_*t*_	1.20*	**-3.32** *	0.00*	0.22*	0.07*	0.19	0.06*	0.56*	0.04*	-0.47*
*DIN*_*t*_	-3.90*	**-1.66**	-0.01*	-0.43*	0.37*	1.86*	-0.10*	0.51*	0.44*	5.78*
*I*_*t*_	0.18*	**-0.18** *	0.00*	0.00	0.00	0.05*	0.00	0.00*	0.00*	0.54*
c) High-volatility period (*s*_*t*_ = 3)										
	Intercept	*r*_*t*-1_	*N*_*t*-1_	*F*_*t*-1_	*P*_*t*-1_	*D*_*t*-1_	*B*_*t*-1_	*T*100_*t*-1_	*DIN*_*t*-1_	*I*_*t*-1_
*r*_*t*_	0.01	**0.12** *	0.00	0.00	0.00	-0.02	0.00	0.01*	0.00	-0.01
*N*_*t*_	5.65*	**-127.10** *	0.90*	-10.34*	7.84*	53.48*	-0.17	-1.37*	-0.24*	-8.42
*F*_*t*_	0.68*	**-1.06** *	0.01*	-0.29*	-0.05	-0.15	0.02	-0.01	-0.01*	0.28*
*P*_*t*_	1.12*	**-1.55** *	-0.01*	-0.07*	-0.19*	0.97*	0.01	0.00	-0.01*	-0.97*
*D*_*t*_	0.13*	**0.04**	0.00	-0.08*	0.05*	0.39*	0.01*	0.00	0.01*	0.08*
*B*_*t*_	2.95*	**1.41**	0.01*	-1.02*	0.69*	6.65*	0.20*	0.09*	0.00	-2.32*
*T*100_*t*_	-0.60*	**-8.24** *	0.02*	-0.46*	0.32*	1.89*	0.05	0.30*	0.01*	1.73*
*DIN*_*t*_	1.31	**5.76**	-0.02*	-0.95*	1.73*	4.36*	-0.32*	0.48*	0.68*	3.38*
*I*_*t*_	0.15*	**-0.06**	0.01*	0.03*	-0.01*	-0.14*	0.00	0.01*	0.01*	0.74*

Autoregressive coefficients (different columns in each panel) and intercepts for each equation (different lines in each panel), in the different regimes (different panels) of the MS-VAR model.

* indicates that the parameter was significant at the 5% level.

**Fig 7:**
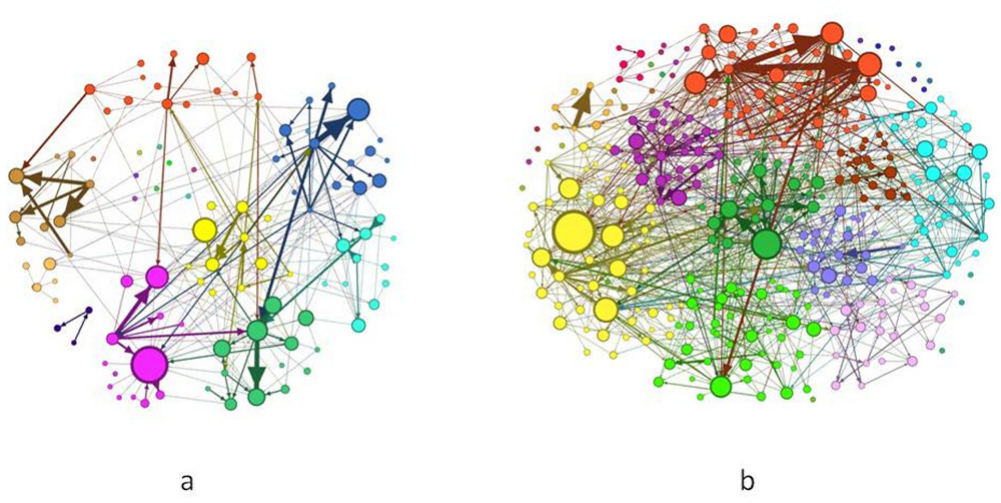
The communication network before (on the left) and after (on the right) a bad news. Left graph shows the communication network on January the 3rd, 2012 and the second graph shows the communication network on January the 4th, 2012. These snapshots were built such that the stronger was the link between two investors, the thicker the graph’s tie was. The node size was proportional to the investor’s in-degree. The difference of node's colour indicates a different modularity class.

Results show that in the initial period of the sample, when the Unicredit stock volatility was at a minimum, the forum network reacted quickly to any change in stock returns. This was found for all the variables considered, i.e., number of investors, turnover, presence of more expert investors and network stability (see *r*_*t*-1_ column in Table 1). Under low volatility, any decrease of stock returns decreased network stability, i.e., there was less persistence of active investors in the forum and less persistent links, higher presence of expert investors and more quality of information shared. Finally, under high volatility, any decrease of stock returns diminished network stability and attracted new investors in the forum compared to the previous day, including but not limited to expert investors (see Fig 7). It is worth noting that during high volatility, bad news had no effect on the quality of information.

Any difference in market volatility did not change the type of market vs. forum relation, except information content of messages. Only the intensity of relations dramatically changed during these situations of high volatility. Results showed that the higher the market volatility, the higher the chance that (good or bad) news had a perturbing effect on the forum network. In cases of bad news, network stability tended to decrease systematically under all volatility regimes, due to more participation and information shared by investors. The turnover of the nodes increased as did tie refresh, due to the tendency of investors to seek new information sources under market pressures. In cases of bad news, the number of expert investors in the forum also systematically increased, independent of volatility.

However, it is interesting to note that investors’ prestige was not influenced by market behaviour during the three volatility periods. This meant that more competent investors were not crucial in channelling communication flows after exogenous market shocks. Market news also did not show any effect on network fragmentation, except during the initial period. On the other hand, the informative content of messages tended to increase after bad news, except in higher volatility situations. This may be similar to when investors were engaged in an interpretation joint-effort that was less analytical and more emotional during periods of external shocks plus uncertainty. Finally, it is worth noting that independent of the market situation, the forum network included information that could help predict stock return behaviour.

## Discussion and Conclusions

Examining information sharing and communication patterns among a large population of investors on an online forum allowed us to look at the phenomenon we called “e-joint attention”. This is a social process through which a multitude of individuals focus on relevant matters collectively, anonymously and at a distance. They do this by combining different interpretations and learning from ‘observing’ each others’ interpretations. E-communication platforms are fundamental in permitting this large scale social process between socially-unrelated individuals. Given the growing diffusion of these platforms, it is essential to understand how relevant knowledge is generated through these decentralised, distributed and self-organised intelligence sources and whether this effort has any concrete learning and market value.

First, we found that market volatility and bad news were the main object of this joint attention process. They triggered a distributed cognitive effort by investors who tried to cope with market uncertainty and external shocks collectively by readapting their network of communication (e.g., [17] and [32]). We found a combination effect between the type of volatility and the type of news especially on the quality of information created through direct and indirect communication. On the one hand, any bad news, independent of the market volatility regimes, increased communication between investors. This confirms previous findings on the investor’s tendency of reacting asymmetrically to bad and good news by increasing information sharing and their sensitivity to social information (e.g., [19], [33] and [20]). On the other hand, while in low market volatility bad news determined higher quality information, under situations of high volatility, any bad news did not have any effect on the quality of message content. This meant that investors reacted more emotionally to external shocks during uncertainty due to the urgency of revising their investment strategies in real-time. In these cases, due to emotions and shortermism the investors considered the opinion of expert investors to be less valuable.

By comparing average network behaviour with real-time adaptation after external market shocks (i.e., our VAR), it is evident that a self-organised, decentralised collective system such as the forum, absorbed uncertainty and generated relevant knowledge in the long-run through e-joint effort attention processes. However, this system was unable to tackle the challenge of providing relevant information in real-time to a multitude of investors during external shocks. We therefore observed these two adaptation paths between the market and the forum.

During general uncertainty and below certain levels of volatility, investors were capable of developing e-joint attention efforts that could predict market behaviour by increasing system connectivity and the informative content of communication. In these cases, the influence of expert investors and their distribution in the system were functional in supporting collective learning. In situations of high volatility and external shocks, investors needing to revise expectations quickly, looked at different sources, which determined unstable patterns of communication. The higher turnover and instability of the relationships between investors negatively impacted information quality, as investors tended to communicate more chaotically. This made experts’ opinions less relevant and the content of their messages less valuable. Furthermore, the prevalence of noise in communication during critical phases indicated that investors were more influenced by pre-existing cognitive bias (e.g., confirmation bias) rather than developing completely new collective interpretations.

To conclude, here we have provided a network aggregate measure of this e-joint attention phenomenon. More in-depth, micro-analyses of socio-cognitive, individual aspects are necessary to understand this social mechanism and verify concrete heuristics used by investors to select information sources and communication partners. Despite this limitation, our results suggest a twofold function of these new e-communication platforms, which could be even ideally be generalized to other online communication platforms. On one hand, there is a positive function that provides dispersed investors (even non-professional) with information and communication opportunities, which might eventually reduce emotional, reactive responses to bad news or panic. The network forum self-organised in recurrent, decentralised organisational patterns that were functional to support investors’ e-joint attention efforts thus providing a learning platform that would be otherwise be hard to access for non-professional investors. However, absorbing volatility trends requires time, i.e., iterated patterns of communication, which were possible under all combinations of volatility and news except when reacting to bad news under high volatility. In these cases, the need for real-time reactions under external shocks in pre-existing situations of uncertainty (i.e., high volatility) made the typical redundancy of these decentralised communication systems dysfunctional. This was due to the prevalence of information noise and marginalised expertise sources.

## Supporting Information

S1 File **Table A**. The network metrics during low and high volatility phases. Values indicate the means of variables. We used a *t-test* to evaluate the statistical significance of the difference between the values. It is worth noting that * indicates that the difference was significant at the 5% level. **Table B**. The network metrics during initial and high volatility phases. The values indicate variables means. We used a *t-test* to evaluate the statistical significance of the differences between values. It is worth noting that * indicates that the difference was significant at 5%. **Table C**. The network metrics during initial and low volatility phases. The values indicate the variable means. We used a *t-test* to evaluate the statistical significance of the difference between the values. It is worth noting that * indicates that the difference was significant at 5%.**Table D**. Summary of the features of our trust schemes. **Table E**. The aggregation of our trust schemes. **Table F**. Test of the trust scheme. **Table G**. Test of the global scheme. **Table H**. Autoregressive coefficients (different columns in each panel) and intercepts for each equation (different lines in each panel), in the different regimes (different panels) of the MS-VAR model.* indicates that the parameter was significant at the 5% level. **Table I**. Model selection following log-likelihood, AIC and BIC. **Table L**. Mean value of the network metrics during the initial period, and the low, moderate and high volatility phases. **Fig A**. Typical performance of the SVM classifier used to classify messages into trading related ones and non-trading related ones. **Fig B**. Unicredit stock log-return, *r*_*t*_, series (blue line, left axis) and the filtered volatility regime *s*_*t|t*_ (red line, right axis) for K = 4.
(DOCX)
