# Correction: Decrypting Financial Markets through E-Joint Attention Efforts: On-Line Adaptive Networks of Investors in Periods of Market Uncertainty

**DOI:** 10.1371/journal.pone.0139528

**Published:** 2015-09-28

**Authors:** Niccolò Casnici, Pierpaolo Dondio, Roberto Casarin, Flaminio Squazzoni

The images for Figs 2, 3 and 5 appear incorrectly. Please view the correct Figs 2, 3 and 5 here.

**Fig 2:**
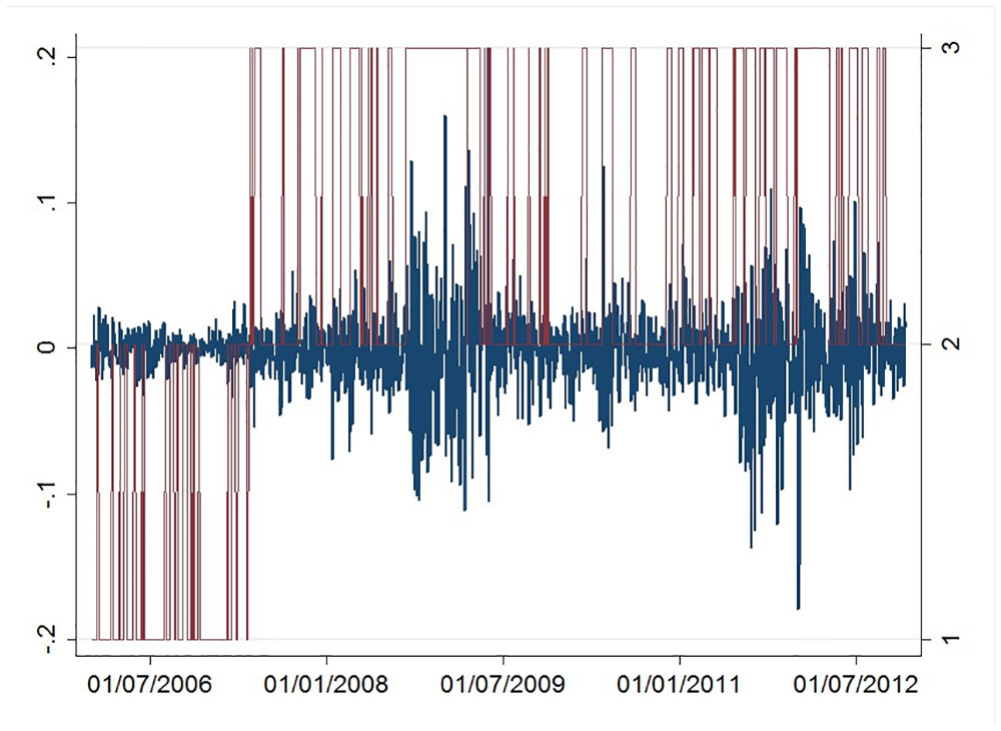
Unicredit stock log-return, *r*_*t*_, series (blue line, left axis) and the filtered regime of volatility *s*_*t|t*_ (red line, right axis).

**Fig 3:**
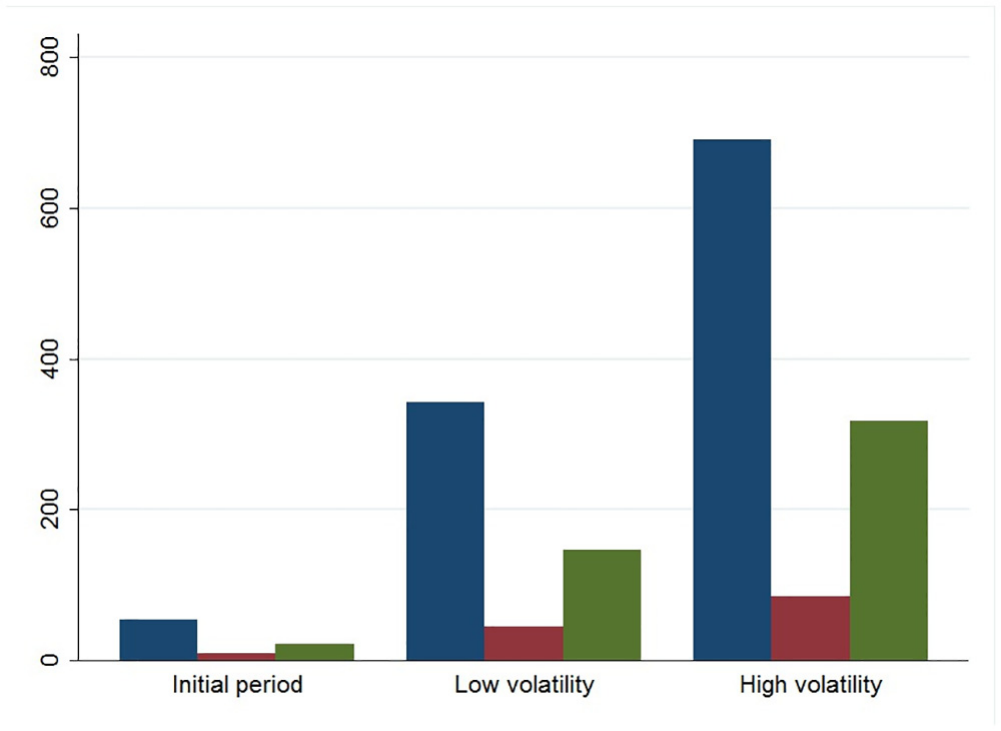
The degree of investors’ participation in the three volatility phases. Blue bars indicate the average number of messages, purple bars show the average number of investors active in the forum, green bars show the average number of ties between the investors.

**Fig 5:**
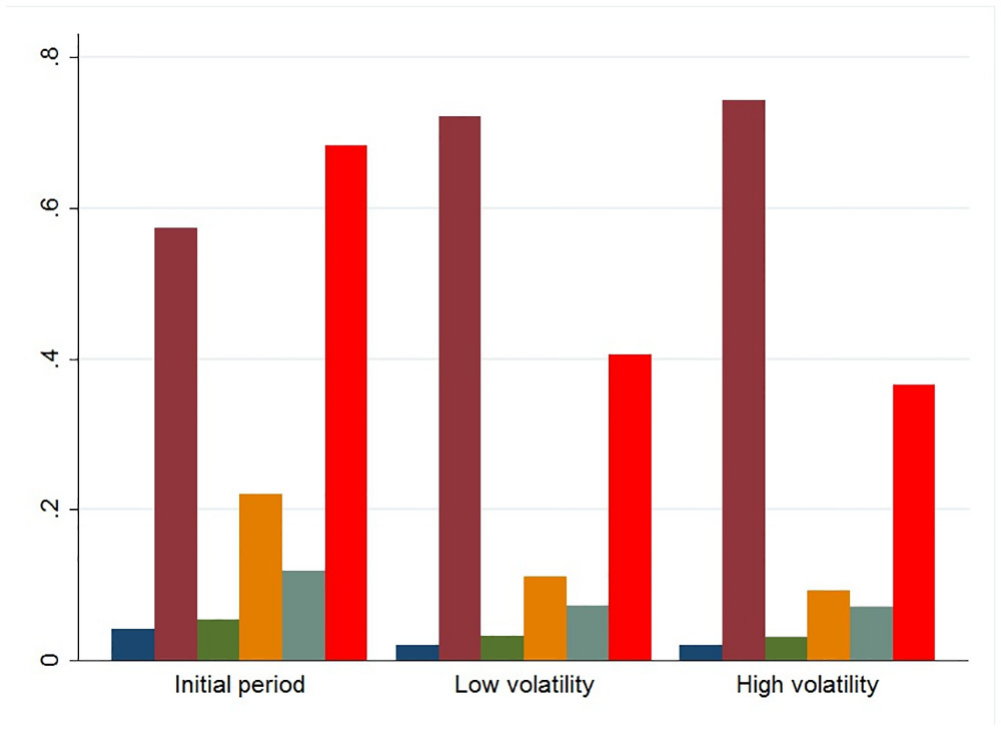
The information content of the messages in the three volatility phases. Blue bars show the average of fundamental analysis messages, purple bars the average of the residual content messages, green bars the average of the news reporting messages, orange bars the average of technical analysis messages, grey bars the average of strategy messages and red bars indicate the average value of the synthetic indicator of financial content.
