# Supplementary material for: Decrypting Financial Markets through E-Joint Attention Efforts: On-Line Adaptive Networks of Investors in Periods of Market Uncertainty
Source: PLoS One. 2015 Aug 5;10(8):e0133712. doi: 10.1371/journal.pone.0133712 (PMC4526688; doi:10.1371/journal.pone.0133712)
Supplement: S1 File — Table A. The network metrics during low and high volatility phases. Values indicate the means of variables. We used a t-test to evaluate the statistical significance of the difference between the values. It is worth noting that * indicates that the difference was significant at the 5% level. Table B. The network metrics during initial and high volatility phases. The values indicate variables means. We used a t-test to evaluate the statistical significance of the differences between values. It is worth noting that * indicates that the difference was significant at 5%. Table C. The network metrics during initial and low volatility phases. The values indicate the variable means. We used a t-test to evaluate the statistical significance of the difference between the values. It is worth noting that * indicates that the difference was significant at 5%.Table D. Summary of the features of our trust schemes. Table E. The aggregation of our trust schemes. Table F. Test of the trust scheme. Table G. Test of the global scheme. Table H. Autoregressive coefficients (different columns in each panel) and intercepts for each equation (different lines in each panel), in the different regimes (different panels) of the MS-VAR model.* indicates that the parameter was significant at the 5% level. Table I. Model selection following log-likelihood, AIC and BIC. Table L. Mean value of the network metrics during the initial period, and the low, moderate and high volatility phases. Fig A. Typical performance of the SVM classifier used to classify messages into trading related ones and non-trading related ones. Fig B. Unicredit stock log-return, r t, series (blue line, left axis) and the filtered volatility regime s t|t (red line, right axis) for K = 4. (DOCX) [file pone.0133712.s001.docx]

Title: Decrypting financial markets through e-joint attention efforts: on-line adaptive networks of investors in periods of market uncertainty

Authors:

Niccolò Casnici

Department of Clinical and Experimental Sciences, University of Brescia, Italy, viale Europa 11, 25123 Brescia, Italy, email: n.casnici@unibs.it

Pierpaolo Dondio

School of Computing, Dublin Institute of Technology, Ireland, Kevin Street, Dublin 8, email: pierpaolo.dondio@dit.ie

Roberto Casarin

Department of Economics, University Ca’ Foscari of Venice, Italy, San Giobbe 873/b, 30121 Venice, Italy, email: [r.casarin@unive.it](mailto:r.casarin@unive.it)

Flaminio Squazzoni

Department of Economics and Management, University of Brescia, Italy, via San Faustino 74/B, 25122 Brescia, Italy, phone number: +39 0302988892, email: flaminio.squazzoni@unibs.it [corresponding author]

Here, is the supplementary material of our work on the *Finanzaonline.com* dataset, including data on average network behaviour under high and low volatility, which complements the data shown in the paper, and details of the model of investors’ expertise. We also included some robustness checks.

**Supporting Information S1**

**Average network behaviour in conditions of high vs. low volatility**

Table A shows the network of investors during low and high volatility periods. Uncertainty increased forum activity in terms of number of nodes, ties and messages. While volatility did not affect network stability, the residual content of messages increased during high uncertainty while the synthetic index of financial content decreased. Despite investors’ tendency to communicate locally more during high volatility periods as more investors were active in the forum, the strength of the division between the communication sub-groups was slightly different. This meant that the fragmentation of the network was both partially and weakly related to market conditions. Finally, the presence of expert investors was higher during the high volatility phases as was their social prestige, i.e., the influence of their opinions on other investors.

|  |  |  |  |  |  |  |  |  |  |
| --- | --- | --- | --- | --- | --- | --- | --- | --- | --- |
|  |  | **Low volatility period** | | | **High volatility period** | | |  |  |
|  |  | Mean | 95% conf. | Interv | Mean | 95% conf. | Interv | diff |  |
|  | *M_t_* | 343.115 | 329.582 | 356.648 | 690.330 | 656.968 | 723.691 | -347.215* |  |
|  | *N_t_* | 45.240 | 43.896 | 46.583 | 85.544 | 81.339 | 89.749 | -40.305* |  |
|  | *G _t_* | 146.841 | 141.094 | 152.587 | 317.851 | 300.251 | 335.451 | -171.011* |  |
|  | *F _t_* | 0.229 | 0.195 | 0.263 | 0.259 | 0.214 | 0.303 | -0.030 |  |
|  | *P _t_* | 0.346 | 0.297 | 0.396 | 0.379 | 0.319 | 0.439 | -0.032 |  |
|  | *D _t_* | 0.339 | 0.333 | 0.345 | 0.349 | 0.343 | 0.354 | -0.010* |  |
|  | *B _t_* | 5.825 | 5.716 | 5.934 | 7.314 | 7.154 | 7.474 | -1.489* |  |
|  | *U _t_* | 0.019 | 0.019 | 0.021 | 0.021 | 0.019 | 0.021 | 0.000 |  |
|  | *W _t_* | 0.033 | 0.031 | 0.034 | 0.031 | 0.029 | 0.032 | 0.002 |  |
|  | *X _t_* | 0.111 | 0.108 | 0.115 | 0.092 | 0.089 | 0.095 | 0.019* |  |
|  | *S _t_* | 0.073 | 0.071 | 0.074 | 0.071 | 0.070 | 0.073 | 0.001 |  |
|  | *C _t_* | 0.722 | 0.717 | 0.727 | 0.743 | 0.738 | 0.747 | -0.020* |  |
|  | *I _t_* | 0.405 | 0.397 | 0.414 | 0.366 | 0.358 | 0.374 | 0.040* |  |
|  | *T100 _t_* | 2.900 | 2.775 | 3.025 | 3.859 | 3.653 | 4.064 | -0.958* |  |
|  | *DIN _t_* | -0.769 | -1.083 | -0.456 | 1.787 | 1.230 | 2.343 | -2.556* |  |
|  |  |  |  |  |  |  |  |  |  |

**S1 Table A.** The network metrics during low and high volatility phases. Values indicate the means of variables. We used a *t-test* to evaluate the statistical significance of the difference between the values. It is worth noting that * indicates that the difference was significant at the 5% level.

Tables B and C show the difference between the initial period and the other two volatility regimes. In both cases, the initial period had less activity, in terms of number of nodes, ties and messages. During the initial period, the turnover of nodes was higher and the refresh of ties was lower. This meant that investors tended to change their communication partners less frequently. During the initial period, there were fewer discussion sub-groups and there was weaker division between them compared to the rest of the series. During the initial period communication and information sharing, messages were more technical and more focused on financial content, as indicated by the synthetic index. Finally, during the initial period, communication patterns involved less expert investors than the rest of the series. Consequently, the social prestige of expert investors was lower, as other investors rarely asked for their opinion.

|  |  |  |  |  |  |  |  |  |  |
| --- | --- | --- | --- | --- | --- | --- | --- | --- | --- |
|  |  | **Initial period** | | | **High volatility period** | | |  |  |
|  |  | Mean | 95% conf. | Interv | Mean | 95% conf. | interv | diff |  |
|  | *M_t_* | 54.690 | 48.677 | 60.703 | 690.330 | 656.968 | 723.691 | -635.640* |  |
|  | *N_t_* | 9.581 | 9.084 | 10.079 | 85.544 | 81.339 | 89.749 | -75.963* |  |
|  | *G _t_* | 21.037 | 19.058 | 23.016 | 317.851 | 300.251 | 335.451 | -296.814* |  |
|  | *F _t_* | 0.451 | 0.350 | 0.552 | 0.259 | 0.214 | 0.303 | 0.192* |  |
|  | *P _t_* | 0.226 | 0.115 | 0.366 | 0.379 | 0.319 | 0.439 | -0.153* |  |
|  | *D _t_* | 0.248 | 0.232 | 0.264 | 0.349 | 0.343 | 0.354 | -0.101* |  |
|  | *B _t_* | 2.756 | 2.638 | 2.873 | 7.314 | 7.154 | 7.474 | -4.559* |  |
|  | *U _t_* | 0.042 | 0.033 | 0.051 | 0.021 | 0.019 | 0.021 | 0.022* |  |
|  | *W _t_* | 0.054 | 0.038 | 0.069 | 0.031 | 0.029 | 0.032 | 0.023* |  |
|  | *X _t_* | 0.221 | 0.207 | 0.235 | 0.092 | 0.089 | 0.095 | 0.129* |  |
|  | *S _t_* | 0.118 | 0.108 | 0.128 | 0.071 | 0.070 | 0.073 | 0.047* |  |
|  | *C _t_* | 0.573 | 0.556 | 0.591 | 0.743 | 0.738 | 0.747 | -0.169* |  |
|  | *I _t_* | 0.683 | 0.640 | 0.725 | 0.366 | 0.358 | 0.374 | 0.317* |  |
|  | *T100 _t_* | 0.031 | 0.010 | 0.052 | 3.859 | 3.653 | 4.064 | -3.828* |  |
|  | *DIN _t_* | -3.158 | -3.524 | -2.792 | 1.787 | 1.230 | 2.343 | -4.944* |  |
|  |  |  |  |  |  |  |  |  |  |

**S1 Table B.** The network metrics during initial and high volatility phases. The values indicate variables means. We used a *t-test* to evaluate the statistical significance of the differences between values. It is worth noting that * indicates that the difference was significant at 5%.

|  |  |  |  |  |  |  |  |  |  |
| --- | --- | --- | --- | --- | --- | --- | --- | --- | --- |
|  |  | **Initial period** | | | **Low volatility period** | | |  |  |
|  |  | Mean | 95% conf. | Interv | Mean | 95% conf. | interv | diff |  |
|  | *M_t_* | 54.690 | 48.677 | 60.703 | 343.115 | 329.582 | 356.648 | -288.425* |  |
|  | *N_t_* | 9.581 | 9.084 | 10.079 | 45.240 | 43.896 | 46.583 | -35.658* |  |
|  | *G _t_* | 21.037 | 19.058 | 23.016 | 146.841 | 141.094 | 152.587 | -125.804* |  |
|  | *F _t_* | 0.451 | 0.350 | 0.552 | 0.229 | 0.195 | 0.263 | 0.222* |  |
|  | *P _t_* | 0.226 | 0.115 | 0.366 | 0.346 | 0.297 | 0.396 | -0.121* |  |
|  | *D _t_* | 0.248 | 0.232 | 0.264 | 0.339 | 0.333 | 0.345 | -0.091* |  |
|  | *B _t_* | 2.756 | 2.638 | 2.873 | 5.825 | 5.716 | 5.934 | -3.069* |  |
|  | *U _t_* | 0.042 | 0.033 | 0.051 | 0.019 | 0.019 | 0.021 | 0.022* |  |
|  | *W _t_* | 0.054 | 0.038 | 0.069 | 0.033 | 0.031 | 0.034 | 0.021* |  |
|  | *X _t_* | 0.221 | 0.207 | 0.235 | 0.111 | 0.108 | 0.115 | 0.110* |  |
|  | *S _t_* | 0.118 | 0.108 | 0.128 | 0.073 | 0.071 | 0.074 | 0.045* |  |
|  | *C _t_* | 0.573 | 0.556 | 0.591 | 0.722 | 0.717 | 0.727 | -0.149* |  |
|  | *I _t_* | 0.683 | 0.640 | 0.725 | 0.405 | 0.397 | 0.414 | 0.277* |  |
|  | *T100 _t_* | 0.031 | 0.010 | 0.052 | 2.900 | 2.775 | 3.025 | -2.869* |  |
|  | *DIN _t_* | -3.158 | -3.524 | -2.792 | -0.769 | -1.083 | -0.456 | -2.388* |  |
|  |  |  |  |  |  |  |  |  |  |

**S1 Table C.** The network metrics during initial and low volatility phases. The values indicate the variable means. We used a *t-test* to evaluate the statistical significance of the difference between the values. It is worth noting that * indicates that the difference was significant at 5%.

**Presence and role of expert investors**

In order to study the presence and role of expert investors in communication patterns, we built a model that included five main features, which measured the level of perceived investors’ trustworthiness: (i) communication activity of investors in the forum, (ii) longevity of their active presence in the forum, (iii) the regularity of their communication activity over time, (iv) the pertinence of information content of their messages, and (v) investors’ influence on the forum. Following [1], for (i), we measured how much an investor communicated on the forum, not considering *when* the activity happened and *what* its content was. At each time *t*, we mapped the number of posts ${(N}_{p}(i,t))$, the number of threads opened $(N_{3D}\left( i,t \right))$ and the total length of the posts by investor *i* ${(L}_{p}\left( i,t \right))$. For (ii), we measured the number of posts for each investor *i,* considering the time interval between the last post $t_{last}$ and first post $t_{first}$. It is important to note that this indicator was pivotal, given that the stock market is a very selective environment and investors’ longevity can implicitly signal their survival capability. Indeed, the forum itself could reflect the survival time of an investor in the real stock market. This idea is corroborated, in the period considered, as there was a sharp decrease in the community in 2007 and 2008, i.e., during the stock market global crisis.

As for (iii), in order to measure how the distribution of each investor’s activity was regular over time *t*, we considered a time interval *Π* (e.g., one day), an activity threshold $\kappa$ (e.g., one message), a time interval $[t_{first},t_{last}]$, with $t_{last}\leq t$, which referred to the interval between the last and the first message posted by investor *i* until time *t*. While the total number of intervals was $N_{tot}=\left( t_{\mathrm{last}}- t_{\mathrm{first}} \right)/\Pi$, we defined the function $A(t_{1}, t_{2})$ that took the value 1 if investor $i$ posted at least $\kappa$messages in the period $[t_{1},t_{2}]$ and the value of 0 otherwise. We then defined the persistency of investor’s communication, i.e., the percentage of intervals where investor *i* was active with at least one message (we used $\kappa=1$), as follows:

$P_{\tau}\left( i,t \right)=\frac{1}{N_{\mathrm{tot}}}\sum_{n=0}^{N_{\mathrm{tot}}} A(n\tau,\left( n+1 \right)\tau)$ (1)

and calculated the daily, weekly, and monthly persistence (respectively, $P_{1}\left( i,t \right)$, $P_{7}\left( i,t \right)$ and $P_{30}\left( i,t \right)$).

In order to measure the pertinence of the investors’ messages (iv), we built an information content metric that included the number of messages with no-null trading-related content posted by investor *i*, called $I\left( i,t \right)$ and the proportion of trading related messages posted by investor *i* over the total number of messages posted by *i*, i.e.

$\bar{I}\left( i,t \right)=\frac{I\left( i,t \right)}{N_{p}(i,t)}$ (2)

The content info metric depends on a text categorization process performed over forum posts. We trained a binary classifier using both a supervised and a semi-supervised approach. We first manually labelled a set of 700 posts, labelling each post as trading related or non-trading related based on their content. A further 300 messages were labelled and used as testing set. A trading related message contains fundamental analysis, technical analysis, market comments, it reports financial news or it contains explicit trading suggestions.

In order to weight how useful was a keyword in the identification of trading-related messages, we used the well-known TF-IDF metrics. A keyword had high TF-IDF value (and therefore is useful for our classification task) if it was very frequent in a trading-related message while it was only rarely present in messages unrelated to trading.

Following a semi-supervised approach similar to [2], we augmented the training and testing set using a specialized financial dictionary provided by *Finanzaonline.com*, reaching the size of 2500 messages for the training set and 800 for the testing set.

We then trained a linear support vector machine classifier (see [3] for an overview of the method) for the binary classification problem of dividing messages into trading related and non-trading related. Fig. A shows the typical performance values of the classifier. The classifier had an accuracy of 83.5%, with similar values for precision and recall. These performance values are coherent with the values reported in literature for similar text categorization problems (e.g., [3]).

Correctly Classified Instances 667 83.5052 %

Incorrectly Classified Instances 133 16.4948 %

Kappa statistic 0.6468

Mean absolute error 0.1755

Root mean squared error 0.3797

Relative absolute error 37.1309 %

Root relative squared error 78.1412 %

Total Number of Instances 800

=== Detailed Accuracy By Class ===

TP Rate FP Rate Precision Recall F-Measure ROC Area Class

0.757 0.117 0.8 0.757 0.778 0.861 0

0.883 0.243 0.855 0.883 0.869 0.861 1

Weighted Avg. 0.835 0.195 0.834 0.835 0.834 0.861

**S1 Fig. A.** Typical performance of the SVM classifier used to classify messages into trading related ones and non-trading related ones.

The assumption was that the higher the content info was, the higher the likelihood would be that the message conveyed relevant trading related information. This allowed us to measure the pertinence of each forum investor, so that high content information increased the trust score of investors. As regards to influence (v), we calculated the number of quotations received by investor *i* during the period $[t-\Delta t,t]$ ($N_{q,\Delta t}(i,t)$), the number of quotations received by investor *i* in the period $[t-\Delta t,t]$ ($N_{u,\Delta t}(i,t)$) and number of quotations per message received by investor *i* during the period $[t-\Delta t,t]$ ($\bar{N_{q,\Delta t}}(i,t)$).

This gave us twelve features ($f$) (see Table D) that were aggregated by treating them as a multi-value logic propositions. Given that these features could be viewed as “vague” expressions that can be experienced at different degrees (for example, an investor could be *highly* active or *not so* regular and so on), we modelled them as ‘fuzzy’ concepts. We first defined a degree of truth for each feature $f_{x}$ for each investor $i$, quantifying how much investor $i$ had the feature $f_{x}$. We defined a membership function $\mu_{f_{x}}:\left[ 0,1 \right]\to[0,1]$, for each feature $f_{x}$ over the percentile score $p_{f_{x}}(i,t)$ of each user *i* at time *t* in the feature $f_{x}$. We used the same shape for all the membership functions $\mu_{f_{x}}$, that is a triangular function with core $[0.5,1]$, modelling the fuzzy term *high*. This meant that, given the percentile score $p_{f_{x}}(i,t)$ of user *i* at time *t* for feature $f_{x}$, the degree of truth $\mu_{f_{x}}\left( i,t \right)$ was as follows:

$\mu_{f_{x}}\left( i,t \right)=\left\{ \begin{matrix} 0 & \mathrm{for} p_{f_{x}}\left( i,t \right)\leq0.5 \\ {2(p}_{f_{x}}\left( i,t \right)-0.5) & \mathrm{elsewhere} \end{matrix}\begin{aligned} \\ \end{aligned} \right.$ (3)

Then, each feature $f_{x}$was aggregated in a final trust value in a two-step process, using the Łukasiewicz logic (e.g., [4]). The AND $\left( \bigwedge\right)$, OR ($\bigvee$) and NOT ($\neg$) operators for the Łukasiewicz logic are defined as follows:

$$a\wedge b=\max\left( a+b-1,0 \right)$$

$$a\vee b=\min\left( a+b,1 \right)$$

$$\neg a=1-a$$

where *a* and *b* are real numbers in [0,1]. In order to generate a final trust value for each investor, first the 12 features identified were aggregate into the following macro-areas: time-based feature, activity-based feature, pertinence related evidence and influence related evidence. The features where aggregated using AND and OR Łukasiewicz operators as described in Table E, generating a degree of truth for each macro-area. Finally, all the degrees of truth of the four macro-areas were aggregated by a logical AND to assign a final trust value for each investor.

| **Feature** | **Text** | **Definition** |
| --- | --- | --- |
| $f_{1}$ | $N_{p}\left( i,t \right)$ | Number of posts (i.e. messages) by investor *i* at time *t* |
| $f_{2}$ | $N_{3D}(i,t)$ | Number of threads opened by investor *i* at time *t* |
| $f_{3}$ | $L_{p}\left( i,t \right)$ | Total length of the posts by investor *i* at time *t* |
| $f_{4}$ | $t_{first}-t_{last}$ | Time between first and last message (longevity metric) |
| $f_{5}$ | $P_{1}(i,t)$ | Daily persistency at time *t* |
| $f_{6}$ | $P_{7}(i,t)$ | Weekly Persistency at time *t* |
| $f_{7}$ | $P_{30}(i,t)$ | Monthly Persistency at time *t* |
| $f_{8}$ | $I(i,t)$ | Number of messages with not-null content info posted by investor *i* at time *t* |
| $f_{9}$ | $\bar{I}\left( i,t \right)$ | Average information content of the messages of investor *i* at time *t* |
| $f_{10}$ | $N_{q,\Delta t}(i,t)$ | Number of quotations received by investor *i* in the period $[t-\Delta t,t]$ |
| $f_{11}$ | $N_{u,\Delta t}(i,t)$ | Number of users received by investor *i* in the period $[t-\Delta t,t]$ |
| $f_{12}$ | $\bar{N_{q,\Delta t}}(i,t)$ | Number of quotation per message received by investor *i* in the period $[t-\Delta t,t]$ |

**Table D.** Summary of the features of our trust schemes.

| **Trust Scheme** | **Aggregation Formula** | **Comment** |
| --- | --- | --- |
| Time-Based - $T_{S_{1}}$ | $f_{4}\wedge(f_{5}\wedge f_{6}\wedge f_{7})$ | We assumed that both high longevity and high regularity at different periods was needed |
| Pertinence- $T_{S_{2}}$ | $f_{8}\bigwedge f_{9}$ |  |
| Influence- $T_{S_{3}}$ | $f_{10}\wedge f_{11}\wedge f_{12}$ |  |
| Activity - ${T_{S}}_{4}$ | $f_{2}\vee(f_{3}\bigwedge f_{1})$ | We assumed that opening a conversation ($f_{2}$) could only increase the trust value. Indeed, as opening a thread could be considered an advanced operation that only a small subset of investors could perform, an AND operation would have penalized very active investors too much. |
| Final Trust Value | $T_{S_{1}}\wedge T_{S_{2}}\wedge T_{S_{3}}\wedge T_{S_{4}}$ |  |

**S1 Table E.** The aggregation of our trust schemes.

As these processes are dynamic, we calculated and updated all variables for each investor monthly. Furthermore, these metrics were tested on *Finanzaonline.com* in July 2009, with a dataset composed by about 9 million messages. We validated our results against an explicit poll published in the *Finanzaonline* forum, asking forum members to identify trustworthy investors. The anonymous poll received almost 300 answers. Results showed a clear consensus about the most trustworthy investors. Following this test, we divided investors into ordered tiers. The first tier included the 10 most trustworthy investors, covering 78.5% of preferences, the second included investors from position 11 to 50 (89% of overall preferences). A trust computation was successful in recognizing tier 1 and tier 2 investors as trustworthy. We evaluated the accuracy of our metric by comparing it with the one obtained from the users’ poll.

We defined the metric as follows:

$E\left( n,t \right)= \frac{1}{n} \sum_{x=1}^{n} |C_{rank}\left( x,t \right)-T_{rank}(x,t)|$ (4)

where $n$ was the number of investors included in the metric up to time *t*, $C_{rank}\left( x,t \right)$ was the ranking of investor *x* according to the community survey, $T_{rank}(x,t)$ was the rank according to our trust calculation. *E(n,t)* measured the average error between our predictions and investors’ opinion for the set of the top-n ranked investors. It is worth noting that *E(10,T)* and *E(50,T)*, with *T* the end of the sample period, were of particular relevance. The metric *E(n,T)* gave the average error generated by our computation of the whole sample (see Tables F and G respectively for each trust scheme and for the global scheme). Results showed that there was an overall very high degree of precision with an average error of 3.4 positions for the top-ten investors using a set of 5,015 investors. Time-based and activity-based schemes were very effective in representing individual trust.

|  | **Trust Scheme** | E(10,T) | E(50,T) |
| --- | --- | --- | --- |
| Time-Based | Longevity | 112 | 400 |
|  | Persistency | 31 | 120 |
|  | Total | 48 | 109 |
| Activity-Based | Activity | 19.6 | 91.8 |
| Pertinence | Pertinence | 55 | 111 |
| Social-Based | Authority | 63 | 198 |

**S1 Table F.** Test of the trust scheme.

|  | E(10,T) | E(50,T) |
| --- | --- | --- |
| Total Value | **3.4** | **40.7** |

**S1 Table G.** Test of the global scheme.

**Robustness check for the MSBVAR**

**Financial market effects**

UniCredit S.p.A. is part of the FTSE MIB Index (previously S&P MIB from 2/6/2003 to 1/6/2009) which is the primary benchmark Index for the Italian equity markets. Its origin traces back to a merger between Borsa Italiana and LSE (London Stock Exchange). Thus, in order to control for the financial market effect, the daily log-return on the FTSE MIB All share market index at time $t-1$, ($b_{t}^{IT}$) was considered as covariate.

Since November 2005, UniCredit ordinary shares were also admitted to listing and trading on the Official Market of the Frankfurt Stock Exchange, whose main Index was DAX. Since December 2007, they were also admitted to listing and trading on the largest Stock Exchange in Central Eastern Europe, the Warsaw Stock Exchange, whose main index was WIG20. In order to control for the effects of these markets, we considered the log-returns on DAX and WIG20 at time t-1, ($b_{t}^{GE}$ and $b_{t}^{PL}$, respectively) as covariates. Therefore, the MSBVAR model with $K=3$ regimes included the following vectors of variables and was defined as

$\left( \begin{matrix} \mathbf{z}_{t} \\ r_{t} \end{matrix} \right)=A\left( s_{t} \right)\mathbf{x}_{t}+B\left( s_{t} \right)\left( \begin{matrix} \begin{matrix} \mathbf{z}_{t-1} \end{matrix} \\ r_{t-1} \end{matrix} \right)+\boldsymbol{\varepsilon}_{t}, \boldsymbol{\varepsilon}_{\mathbf{t}}\sim N_{n}(\mathbf{0},\Sigma(s_{t}))$ (5)

with:

$$A\left( s_{t} \right)=\sum_{k=1}^{K} A_{k}I(s_{t}=k), B\left( s_{t} \right)=\sum_{k=1}^{K} B_{k}I(s_{t}=k), \Sigma\left( s_{t} \right)=\sum_{k=1}^{K} \Sigma_{k} I(s_{t}=k)$$

and

$\mathbf{x}_{t}=(1,b_{t}^{IT},b_{t}^{GE},b_{t}^{PL})'$ and $\mathbf{z}_{t}=(N_{t},F_{t},P_{t},D_{t},B_{t},T{100}_{t},DIN_{t},I_{t})'$

Table H shows that most of the results obtained with the model with one exogenous variable can be generalised after including other two control variables, i.e., the Frankfurt and Warsaw markets.

| 1. Initial period ${(s}_{t}=1)$ | | | | | | | | | | | | | | | | | | |  |  |  |
| --- | --- | --- | --- | --- | --- | --- | --- | --- | --- | --- | --- | --- | --- | --- | --- | --- | --- | --- | --- | --- | --- |
|  | Intercept | | | $r_{t-1}$ | $N_{t-1}$ | $F_{t-1}$ | $P_{t-1}$ | | $D_{t-1}$ | | $B_{t-1}$ | | $T{100}_{t-1}$ | | $DIN_{t-1}$ | | $I_{t-1}$ | |  |  |  |
| $r_{t}$ | 0.00 | | | **0.19*** | 0.00 | 0.00 | 0.00 | | 0.00 | | 0.00 | | 0.00 | | 0.00 | | 0.00 | |  |  |  |
| $N_{t}$ | 4.47* | | | **-9.11*** | 0.57* | 0.29* | -0.01 | | -3.53* | | 0.12* | | -0.22* | | -0.07* | | 0.02 | |  |  |  |
| $F_{t}$ | 0.78* | | | **-4.60*** | -0.04* | -0.07* | -0.13* | | -0.70* | | 0.06* | | -0.19* | | 0.01* | | 0.11 | |  |  |  |
| $P_{t}$ | 0.39* | | | **0.67*** | -0.01* | 0.02 | -0.13* | | -0.88* | | -0.05* | | -0.03* | | -0.01* | | 0.33é | |  |  |  |
| $D_{t}$ | 0.18* | | | **0.66*** | 0.00 | 0.00 | 0.01* | | 0.11* | | 0.00 | | -0.02* | | 0.01* | | 0.02* | |  |  |  |
| $B_{t}$ | 1.50* | | | **-0.94*** | 0.10* | 0.07 | -0.02* | | -0.37* | | 0.07* | | -0.03* | | -0.02* | | 0.33* | |  |  |  |
| $T{100}_{t}$ | 0.01* | | | **-2.85*** | -0.01* | 0.03 | 0.02 | | -0.04* | | 0.05* | | 0.24* | | -0.01* | | -0.03 | |  |  |  |
| $DIN_{t}$ | -0.04 | | | **0.57*** | -0.10* | -0.10* | 0.04* | | 2.32* | | -0.05* | | -0.91* | | 0.64* | | -0.84* | |  |  |  |
| $I_{t}$ | 0.57* | | | **-1.12*** | 0.00 | -0.02 | 0.03* | | 0.16 | | -0.04* | | -0.07* | | 0.00 | | 0.29* | |  |  |  |
| 1. Low-volatility period ${(s}_{t}=2)$ | | | | | | | | | | | | | | | | | | |  |  |  |
|  | Intercept | | | $r_{t-1}$ | $N_{t-1}$ | $F_{t-1}$ | $P_{t-1}$ | | $D_{t-1}$ | | $B_{t-1}$ | | $T{100}_{t-1}$ | | $DIN_{t-1}$ | | $I_{t-1}$ | |  |  |  |
| $r_{t}$ | -0.07* | | | **-0.01*** | 0.01* | 0.00 | 0.01 | | 0.01* | | 0.00 | | 0.00 | | 0.01* | | -0.02* | |  |  |  |
| $N_{t}$ | 82.34* | | | **-8.01** | 0.75* | -1.75* | 1.56* | | 0.06 | | 0.62* | | -0.27* | | 0.00 | | -8.71* | |  |  |  |
| $F_{t}$ | 1.37* | | | **-1.32*** | -0.07* | 0.00 | 0.06 | | -0.22* | | -0.63* | | 0.00 | | -0.01 | | 0.00* | |  |  |  |
| $P_{t}$ | 0.45* | | | **-0.98*** | 0.00* | 2.04* | -0.06* | | -0.03 | | -0.44* | | -0.07 | | 0.00 | | -0.01 | |  |  |  |
| $D_{t}$ | -0.01 | | | **-0.44*** | 0.00 | 95.20* | 0.12 | | 0.00* | | -0.02 | | 0.01* | | 0.27* | | 0.00 | |  |  |  |
| $B_{t}$ | 0.03 | | | **0.07** | 0.00* | -0.42* | 18.34* | | -2.25 | | 0.03* | | -0.20* | | 0.15* | | 1.21* | |  |  |  |
| $T{100}_{t}$ | 0.07* | | | **-0.01** | -0.01* | 0.67* | 6.22* | | 341.52* | | -2.78* | | 0.00 | | 0.27* | | 0.01 | |  |  |  |
| $DIN_{t}$ | 1.76* | | | **0.63*** | 0.02* | 0.01 | -7.46* | | 15.09* | | 58.14* | | -1.23* | | 0.04 | | -0.43* | |  |  |  |
| $I_{t}$ | 0.00 | | | **-0.40*** | 0.00* | 0.52* | 0.55* | | 0.27* | | 0.41* | | -0.32* | | -0.03* | | 0.01* | |  |  |  |
| 1. High-volatility period $(s_{t}=3)$ | | | | | | | | | | | | | | | | | | | | |  |
|  | | | Intercept | $r_{t-1}$ | | $N_{t-1}$ | $F_{t-1}$ | | $P_{t-1}$ | | $D_{t-1}$ | | $B_{t-1}$ | | $T{100}_{t-1}$ | | $DIN_{t-1}$ | | $I_{t-1}$ | | |
| $r_{t}$ | | | 0.25 | **0.00*** | | 0.00 | 0.00 | | -0.01 | | 0.00 | | 0.00 | | 0.00* | | -0.01 | | -0.27* | | |
| $N_{t}$ | | | 19.40* | **-93.00*** | | 0.77* | -17.84* | | 9.58* | | 5.90* | | -0.85* | | -0.71* | | -0.16* | | 2.49 | | |
| $F_{t}$ | | | 1.26* | **-2.70*** | | 0.01 | 0.00 | | -0.06 | | -0.11 | | -0.19* | | -0.02 | | -0.01 | | 0.01* | | |
| $P_{t}$ | | | 0.51* | **-2.80*** | | -1.07* | -2.07* | | 0.00 | | 0.22* | | -0.34* | | 0.22* | | -0.03 | | -0.02* | | |
| $D_{t}$ | | | 0.00 | **1.47*** | | 0.03* | -0.86* | | -0.04 | | 0.00* | | -0.03* | | 0.01 | | 0.05* | | 0.00 | | |
| $B_{t}$ | | | 0.07* | **0.88*** | | 0.00 | -0.07* | | 1.22* | | 0.84* | | 0.15* | | 0.08* | | 0.07 | | -5.25* | | |
| $T{100}_{t}$ | | | 0.01 | **-4.53*** | | 0.01* | 2.17* | | 2.37* | | 6.40* | | -4.59* | | 0.01* | | 0.05 | | 0.35* | | |
| $DIN_{t}$ | | | 2.06* | **0.31*** | | 0.00 | 1.58* | | 5.06* | | 4.64* | | -0.69* | | 0.30* | | 0.80* | | 1.13* | | |
| $I_{t}$ | | | 0.18* | **-0.01** | | 0.00 | 0.05* | | 0.04* | | -0.28* | | -0.01 | | 0.00* | | 0.00* | | 0.24* | | |

**S1 Table H.** Autoregressive coefficients (different columns in each panel) and intercepts for each equation (different lines in each panel), in the different regimes (different panels) of the MS-VAR model.* indicates that the parameter was significant at the 5% level.

**Choice of the number of regimes**

In order to select the number of regimes, we applied a model selection criterion based on the marginal likelihood (see Table I). Increasing the number of regimes (first column) implied to increase the marginal log-likelihood (second column) and the number of parameters (third column). Note that the number of parameters to estimate, $((n^{2}+2n+n(n+1)/2)K +K^{2})$, was a polynomial function of the third order with respect to the number of equations (n) and the number of regimes ($K$). The power increase in the number of parameters can produce overfitting and loss of efficiency. This required the use of parsimonious models. For this reason, we applied a goodness of fit indicator that favoured model parsimony, i.e., the Akaike Information Criterion (AIC) and the Bayesian Information Criterion (BIC). The BIC contains a penalty term for adding extra parameters which grows with the sample size. This ensured that, asymptotically, we could not select a larger model over a correctly specified parsimonious one. Following AIC (columns four), the optimal number of regimes was K=4. Note, however, that in large samples, as in our case, the AIC is expected to select the less parsimonious model about 16 percent of the time. Following the BIC (column five), we concluded that the optimal number of regimes was $K=3$.

| **Number of regimes (K)** | **Log-Likelihood** | **Number of parameters** | **AIC** | **BIC** |
| --- | --- | --- | --- | --- |
| 2 | -7802.886 | 292 | 16189.77 | 16843.65 |
| 3 | -7206.502 | 441 | 15295 | 16282.54 |
| 4 | -6902.415 | 592 | 14988.83 | 16314.5 |
| 5 | -6807.881 | 745 | 15105.76 | 16774.04 |

**S1 Table I.** Model selection following log-likelihood, AIC and BIC.

Given that the paper presented only results of the 3-regimes model, here we completed the analysis by reporting results of the 4-regimes model, which would have been selected when applying AIC. Fig. B shows the filtered hidden states (red line). Also in this case, when fitting the MSVAR model, the first regime was associated to the first part of the sample, i.e., the initial period. Thanks to the identification constrain on the variance parameters, the initial period was the one with the lowest variance level, whereas regimes from 2 to 4 were characterized by an increasing level of variance. Tab. L shows the average values of the network metrics in the 4-regimes model. In general, results here corroborated previous results of the 3-regimes model. However, we found a few differences for the variables $T{100}_{t}$ and $DIN_{t}$. In these two cases, the 4 regimes model allowed to provide more detail on the presence and the structural prestige of the expert investors, which did not affect the previous findings.


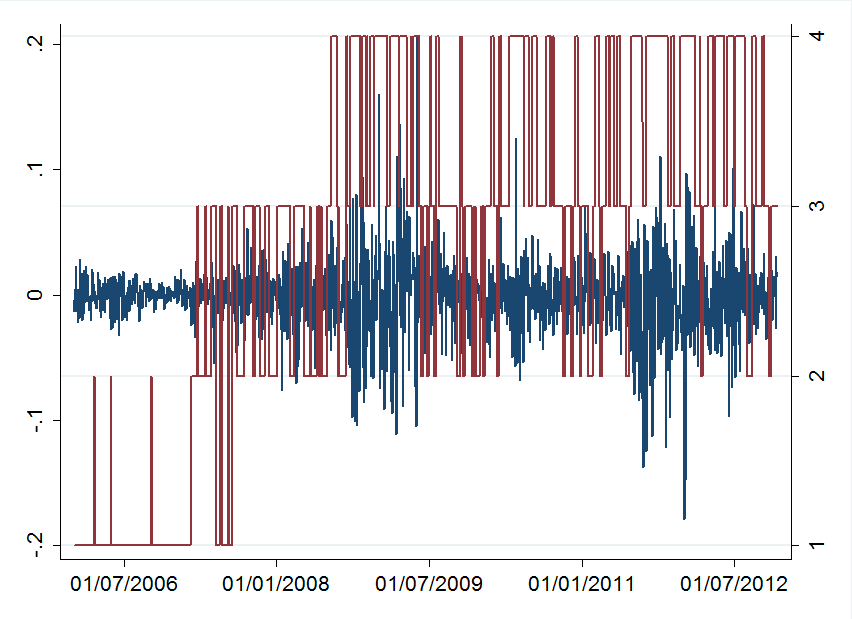


**S1 Fig. B.** Unicredit stock log-return, $r_{t}$, series (blue line, left axis) and the filtered volatility regime $s_{t|t}$ (red line, right axis) for K=4.

|  | **Initial period** | **Low volatility period** | **Moderate volatility period** | **High volatility period** |
| --- | --- | --- | --- | --- |
| *M_t_* | 77.2 | 269.3 | 433.2 | 703.9 |
| *N_t_* | 12.1 | 38.3 | 55.1 | 87.3 |
| *G _t_* | 31.36 | 113.72 | 186.99 | 324.78 |
| *F _t_* | 0.4 | 0.28 | 0.25 | 0.22 |
| *P _t_* | 0.22 | 0.41 | 0.38 | 0.33 |
| *D _t_* | 0.26 | 0.34 | 0.35 | 0.35 |
| *B _t_* | 3.06 | 5.39 | 6.3 | 7.43 |
| *U _t_* | 0.04 | 0.02 | 0.02 | 0.02 |
| *W _t_* | 0.05 | 0.04 | 0.03 | 0.03 |
| *X _t_* | 0.2 | 0.14 | 0.1 | 0.08 |
| *S _t_* | 0.11 | 0.08 | 0.07 | 0.07 |
| *C _t_* | 0.59 | 0.69 | 0.74 | 0.75 |
| *I _t_* | 0.64 | 0.47 | 0.38 | 0.34 |
| *T100 _t_* | 0.39 | 2.87 | 3.17 | 3.84 |
| *DIN _t_* | -2.49 | 2.3 | 0.58 | -1.25 |

**S1 Table L.** Mean value of the network metrics during the initial period, and the low, moderate and high volatility phases.

**References**

1. Dondio, P., Barrett, S., Weber, S., & Seigneur, J. M. Extracting trust from domain analysis: A case study on the Wikipedia project, *Autonomic and Trusted Computing* (pp. 362-373). Springer Berlin Heidelberg (2006).

2. Li, S., Wang, Z., Zhou, G., and Lee, Semi-supervised learning for imbalanced sentiment classification, *IJCAI Proceedings of the Twenty Second International Joint Conference on Artificial Intelligence*, *Barcelona (Spain)*, **22**, 1826-1831 (July 16-22, 2011).

3. Joachims, T. Text categorization with Support Vector Machines: Learning with many relevant features, *Machine Learning: ECML-98, Tenth European Conference on Machine Learning*, *Chemnitz (Germany),* 137-142 (April 21-23, 1998).

4. Lukasiewicz, T., Straccia, U. Managing Uncertainty and Vagueness in Description Logics for the Semantic Web, *Journal of Web Semantics*, **6**, 291-308 (2008).
